# Association between seizures after ischemic stroke and stroke outcome: A systematic review and meta-analysis

**DOI:** 10.1097/MD.0000000000004117

**Published:** 2016-07-08

**Authors:** Tao Xu, Shu Ou, Xi Liu, Xinyuan Yu, Jinxian Yuan, Hao Huang, Yangmei Chen

**Affiliations:** Department of Neurology, The Second Affiliated Hospital of Chongqing Medical University, Chongqing, China.

**Keywords:** Ischemic stroke, Post-ischemic stroke seizures, Mortality, Disability

## Abstract

A systematic review and meta-analysis were performed to investigate a potential association between post-ischemic stroke seizures (PISS) and subsequent ischemic stroke (IS) outcome.

A systematic search of two electronic databases (Medline and Embase) was conducted to identify studies that explored an association between PISS and IS outcome. The primary and secondary IS outcomes of interest were mortality and disability, respectively, with the latter defined as a score of 3 to 5 on the modified Rankin Scale.

A total of 15 studies that were published between 1998 and 2015 with 926,492 participants were examined. The overall mortality rates for the patients with and without PISS were 34% (95% confidence interval [CI], 27–42%) and 18% (95% CI, 12–23%), respectively. The pooled relative ratio (RR) of mortality for the patients with PISS was 1.97 (95% CI, 1.48–2.61; *I*^2^ = 88.6%). The overall prevalence rates of disability in the patients with and without PISS were 60% (95% CI, 32–87%) and 41% (95% CI, 25–57%), respectively. Finally, the pooled RR of disability for the patients with PISS was 1.64 (95% CI, 1.32–2.02; *I*^2^ = 66.1%).

PISS are significantly associated with higher risks of both mortality and disability. PISS indicate poorer prognoses in patients experiencing IS.

## Abstract 2

Supplemental Digital Content is available in the text

## 1 Introduction

The reported incidence of post-ischemic stroke seizures (PISS) currently ranges from 3% to 17%.^[1,2]^ PISS can cause neuronal cell death and can lead to excitotoxicity that induces mitochondrial dysfunction, alterations in cytokine levels, and oxidative stress.^[3,4]^ Moreover, these conditions can have a considerable pathophysiological impact on ischemic stroke (IS) progression to worsen patient prognosis.^[5]^

Recently, the association between PISS and subsequent IS outcome has been of interest, particularly in light of inconsistent data regarding IS outcome where higher mortality and worse functional outcomes have been reported in some studies and not in other studies. These differences are potentially due to differences demographics, study design, time-based definitions of PISS, and time of follow-up that characterize these studies.

Therefore, the goal of the present study was to evaluate the outcome of patients experiencing PISS by conducting a systematic review and meta-analysis.

## 2 Methods

A systematic review and meta-analysis were conducted according to the guidelines previously published for a meta-analysis of observational studies in epidemiology.^[6]^

### 2.1 Study selection

Both the Medline and Embase electronic databases were searched using predefined terms and search criteria. The inclusion criteria for the meta-analysis were: publication in the English language; a cohort, case-control, or cross-section study design; reporting of epidemiologic evidence regarding the outcome of patients with PISS relative to a representative cohort without PISS; reporting of mortality or disability outcomes; assessment of disability after PISS according to the modified Rankin Scale^[7]^; and original research with full-text available. Non-original articles, articles with insufficient data or irrelevant outcomes, and case reports were excluded. There were no restrictions on the time of publication. Four authors (T.X., S.O., H.H., and X.L.) independently evaluated the retrieved studies according to the selection criteria. Discrepancies were resolved by discussion until consensus was reached.

### 2.2 Data extraction and quality assessment

The following data were extracted from each study (by T.X., J.Y., and X.Y.): first author, publication year, population demographics, methods of assessing PISS and IS, information regarding IS outcomes (e.g., mortality rate, prevalence of disability, hazard ratio [HR], risk ratio [RR], odds ratio [OR], and raw data to calculate relative risk [RR]). Statistical adjustments for the main confounding factors of interest (e.g., sex, age distribution, onset time of PISS, time of follow-up, life styles, comorbidities, and baseline stroke severity [BSS]) were also recorded. BSS was confirmed based on clinical features and stroke assessment scale scoring performed upon hospital admission for IS onset.

The primary and secondary outcomes of interest were mortality and disability, respectively, with the latter defined as a score of 3 to 5 on the modified Rankin Scale. According to criteria established by the International League Against Epilepsy, early-onset seizures (ES) were defined as seizures occurring within 7 days after IS onset, and late-onset seizures (LS) were defined as seizures occurring after this period.^[8]^ While this same 7 d interval has been used in other studies, other intervals have been defined for ES and LS, including length of hospitalization.^[9,10]^ Thus, in addition to evaluating an interval of 7 days for defining ES and LS, we also considered ES as those occurring within the period of hospitalization after IS onset, and considered LS as seizures after this period.^[2,8]^ When the occurring time of PISS was not indicated, this was reported in association with the corresponding data. Patients with and without PISS were classified as the PISS group and the non-PISS group, respectively.

A 9-star system based on the Newcastle-Ottawa scale^[11]^ was used to assess study quality.

### 2.3 Statistical analysis

Overall mortality and prevalence of disability in the PISS group versus the non-PISS group were assessed, and RR was used as the summary measure for the association between PISS and subsequent IS outcome. Subgroup analyses were conducted according to the main confounding factors of interest (listed above). To assess between-study heterogeneity, the Cochrane *Q* statistic was calculated. The *I*^2^ statistic was used to quantify magnitude.^[12]^ When statistically significant heterogeneity was absent (*I*^2^ < 50%), the pooled estimate and 95% confidence intervals (CIs) were calculated with a fixed-effects model. Conversely, when significant heterogeneity was present (*I*^2^ ≥ 50%), a random-effects model was used to calculate the pooled estimate. The meta-analysis was extended to a meta-regression to investigate between-study variance (*R*^2^) to quantify the degree of heterogeneity.^[13]^ In addition, the percentage of *R*^2^ was used to depict the level of explained heterogeneity of the variables. A *P* value less than 0.05 was considered statistically significant. Publication bias was investigated visually with funnel plots and statistically with Begg test.^[14]^ STATA version 12.0 (StataCorp, College Station, TX) was used for the statistical analyses performed.

### 2.4 Ethical statement

As all analyses were based on previously published studies, ethical approval was not necessary.

## 3 Results

### 3.1 Study selection

**Figure 1:**
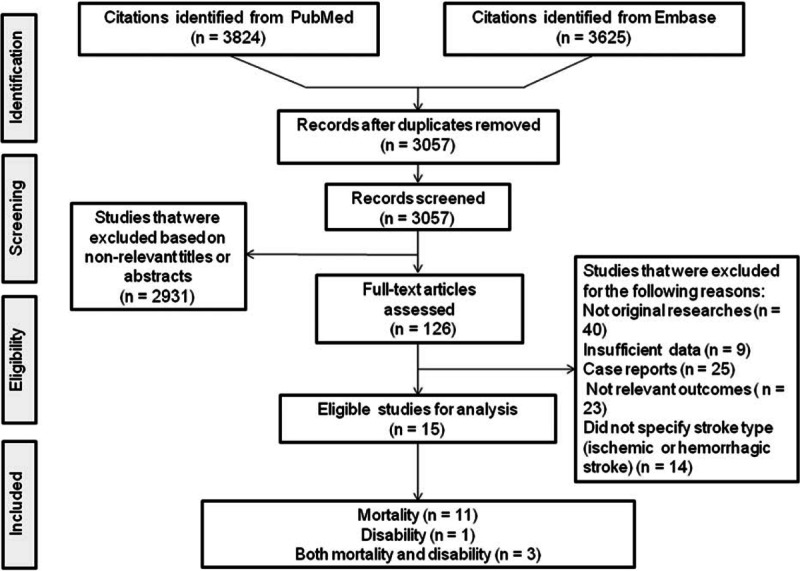
Flowchart of the literature search performed.

Of the 3057 unduplicated records that were identified during our initial search of the Medline and Embase databases, the full texts of 126 articles were reviewed. A total of 15 articles met the inclusion criteria established for this study. Of the included studies, 11 described an association between PISS and mortality,^[15–25]^ one described an association between PISS and disability,^[26]^ and 4 articles described both mortality and disability in patients with PISS^[9,10,27]^ (Fig. 1).

### 3.2 Description of studies and qualitative assessment

A total of 15 studies published between 1998 and 2015 that included 926,492 participants were examined in our meta-analysis. Three main diagnostic approaches for evaluating PISS were used: objective standard criteria based on previously published guidelines (n = 8); International Classification of Diseases (ICD) codes or medical records in databases (n = 1); and clinician-reported diagnosis (n = 6). In addition, 73% (n = 11) of the included studies reported summary data on participant age, with the mean/median ages reported ranging from 8 to 77 years. The BSS for the patients with IS, as assessed by the National Institutes of Health Stroke Scale, the Canadian Neurological Scale, or clinical findings, was reported in 53% (n = 8) of the studies examined. Sixty-seven percent (n = 10) of the included studies also reported information regarding comorbidities, which predominantly included hypertension, diabetes mellitus, and cardiac diseases. In addition, 47% (n = 7) of the included studies adjusted for age, sex, life styles, and differences in comorbidities and BSS between the PISS and non-PISS groups when analyzing the outcomes of interest. A detailed description of these studies is provided in Supplemental Content (Table S1).

Quality scores of the included studies are also listed in Supplemental Content (Table S2). The mean score of the included studies was 5.93 (standard deviation: 1.83; range: 3–9).

### 3.3 Association between PISS and mortality

**Figure 2:**
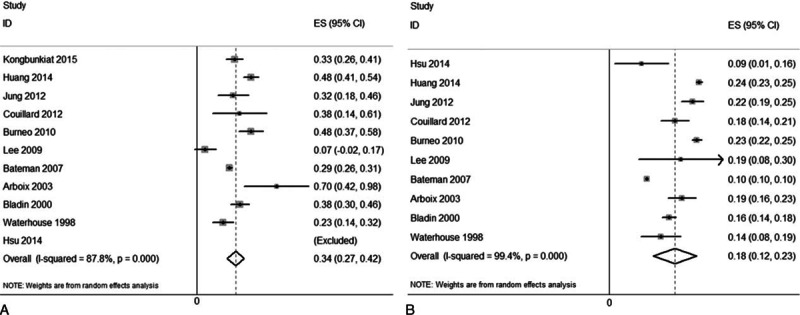
Forest plots of the overall mortality in patients with (A) and without (B) PISS. PISS = post-ischemic stroke seizures.

**Figure 3:**
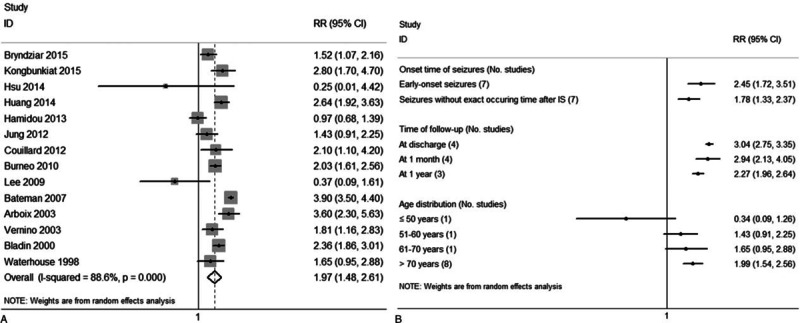
Forest plots of the association between PISS and risk of mortality (A) and the subgroup analyses that were performed to investigate the association between PISS and risk of mortality (B). PISS = post-ischemic stroke seizures.

The overall mortality rates for the patients with (Fig. 2A) and without (Fig. 2B) PISS were 34% (95% CI, 27–42%) and 18% (95% CI, 12–23%), respectively. The pooled RR was 1.97 (95% CI, 1.48–2.61; *I*^*2*^ = 88.6%) for those with PISS compared with those without PISS (Fig. 3A). Correspondingly, in subgroup analyses, a greater risk of mortality was associated with ES (RR, 2.45; 95% CI, and 1.72–3.51). Moreover, mortality was also elevated in the group of patients with PISS that did not have an exact onset time for the PISS reported (RR, 1.78; 95% CI, 1.33–2.37). However, no study was found to report an association between LS and morality in patients with IS. The risk of death in patients with PISS was highest at discharge (RR: 3.04, 95% CI: 2.75–3.35), and then the risk gradually decreased (RR at 1 month after IS onset: 2.94, 95% CI: 2.13–4.05), yet remained high up to 1 year after the onset of IS (RR: 2.27, 95% CI: 1.96–2.64). When age distribution was analyzed, risk of death increased with age and was highest in patients older than 70 years (RR: 1.99, 95% CI: 1.54–2.56) (Fig. 3B). In subgroup analyses, the pooled RR of the studies with no difference in BSS between their PISS and non-PISS groups (RR: 2.92; 95% CI, 1.74–4.89), and of the studies where BSS was more severe in the PISS group than the non-PISS group (RR: 2.15; 95% CI, 1.76–2.62), were statistically significant. Similarly, the pooled RR of the studies with no differences in comorbidities between the PISS and non-PISS groups (RR: 1.65; 95% CI, 1.14–2.38), of the studies with fewer comorbidity events in the PISS group (RR: 2.84; 95% CI, 1.50–5.38), and of the studies with more comorbidity events in the PISS group (RR: 2.10; 95% CI, 1.07–4.10) were all statistically significant. For the confounders of interest, the pooled RR of the adjusted estimates was 2.08 (95% CI, 1.27–3.39) (Table 1).

**Table 1:**
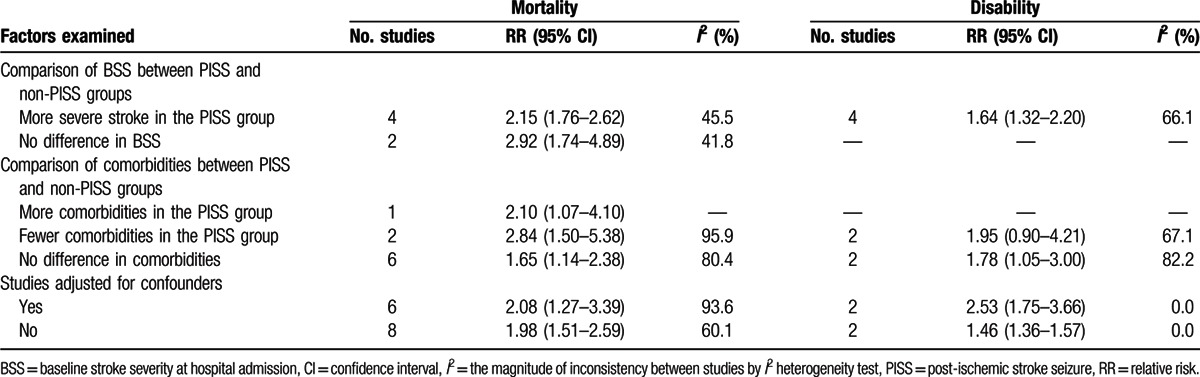
Stratified analyses of the pooled RRs of factors potentially affecting post-ischemic stroke seizures in regard to mortality and disability.

### 3.4 Association between PISS and disability

**Figure 4:**
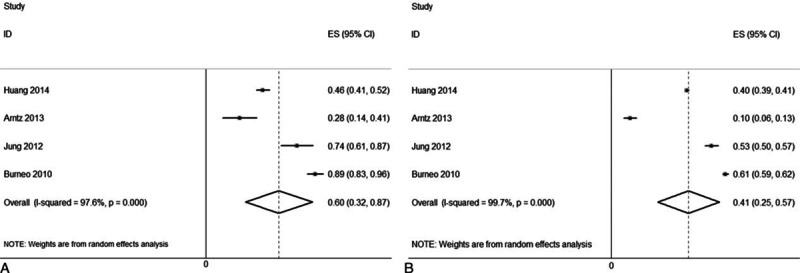
Forest plots of the overall prevalence of disability in patients with (A) and without (B) PISS. PISS = post-ischemic stroke seizures.

**Figure 5:**
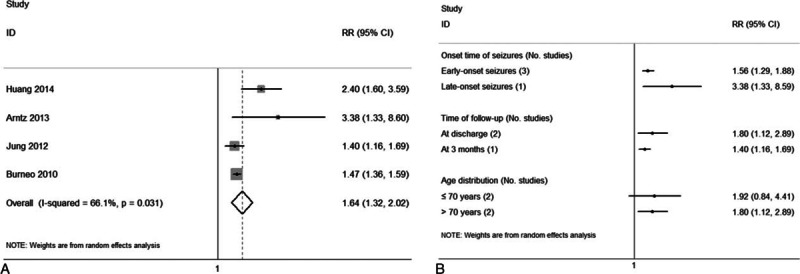
Forest plots of the association between PISS and risk of disability (A) and the subgroup analyses that were performed to investigate the association between PISS and risk of disability (B). PISS = post-ischemic stroke seizures.

The overall prevalence of disability in the patients with (Fig. 4A) and without (Fig. 4B) PISS was 60% (95% CI, 32–87%) and 41% (95% CI, 25–57%), respectively. The pooled RR of disability was 1.64 (95% CI, 1.32–2.02; *I*^2^ = 66.1%) for those with PISS compared with those without PISS (Fig. 5A).

Subgroup analyses suggested that both ES (RR: 1.56; 95% CI, 1.29–1.88) and LS (RR: 3.38; 95% CI, 1.33–8.59) were associated with a greater risk of disability. In particular, the risk of disability was significantly elevated at discharge (RR: 1.80; 95% CI, 1.12–2.89) and at 3 months after IS onset (RR: 1.40; 95% CI, 1.16–1.69). The risk of disability also significantly increased in patients older than 70 years (RR: 1.80; 95% CI, 1.12–2.89), and not in patients younger than 70 years (RR: 1.92; 95% CI, 0.84–4.41) (Fig. 5B). The pooled RR of studies with no differences in comorbidities between the PISS and non-PISS groups was 1.78 (95% CI, 1.05–3.00). While BSS was more severe in the PISS group than in the non-PISS group in all four studies,^[9,10,26,27]^ the pooled RR of the adjusted estimates (2.53) was statistically significant (95% CI, 1.75–3.66). These results suggest that a positive association exists between PISS and risk of disability (Table 1).

### 3.5 Heterogeneity assessment

Due to significant heterogeneity among the included studies, meta-regression analysis was used to explore potential sources of heterogeneity. When mortality of the patients with PISS was analyzed, differences in age distribution and comorbidities between the PISS and non-PISS groups were identified and they accounted for 41.10% and 18.62% of the observed heterogeneity among the included studies, respectively. Finally, the analysis of disability in the patients with PISS identified differences in adjustment for the main confounders of interest, and this accounted for the main heterogeneity observed (approximately 100%) among the included studies.

### 3.6 Publication bias

The funnel plot for the pooled RR appeared asymmetric by visual inspection. However, Begg tests showed no significant evidence of publication bias in our meta-analysis (Supplemental Content: Figure S1 and S2).

## 4 Discussion

To the best of our knowledge, this is the first meta-analysis to investigate possible association(s) between PISS and clinical outcomes. Overall, PISS were found to be associated with increased risks of mortality and disability, thereby indicating poorer prognoses.

IS is a leading cause of seizures in adults, especially in aging populations.^[9,18]^ PISS are a pathological sequelae of hypoxia-ischemia brain injury and they reflect a state of hyperexcitability in brain circuits and abnormally excessive depolarization of individual neurons.^[3,28,29]^ Based on studies that have been conducted using animal models, electrophysiological events appear to play a key role in the progression of brain injury from primary ischemic regions to adjacent regions of secondary injury.^[29]^ PISS have also been found to substantially influence animal survival.^[4]^ Therefore, PISS are not only a consequence of hypoxia-ischemia brain injury, but can also contribute to worsened outcome following hypoxia-ischemia.^[4]^ Consistent with this previously published evidence, our meta-analysis of observational studies indicates that patients with PISS have higher risks of death and disability than those without PISS.

Several studies have reported that PISS generally affect patients that have experienced severe IS, with more severe BSS and a greater frequency of comorbidities being the main factors that contributed to the poor prognoses observed.^[19,30,31]^ However, in the present meta-analysis, pooled estimates of the studies with no statistically significant differences in BSS and comorbidities between the PISS and non-PISS groups suggest that PISS are significantly associated with higher risks of mortality and disability. Furthermore, the pooled adjusted estimates support this association. Thus, our meta-analysis suggests that PISS are independently associated with poorer prognoses in IS patients.

Classifications of PISS have differed among previous studies, mostly in relation to the use of different time-based definitions of IS-related seizures.^[2]^ Consequently, the association between PISS and IS outcome has been unclear. To address this problem, subgroup analyses were conducted as part our meta-analysis. Mortality was found to be significantly elevated in IS patients that experienced ES. The prevalence of disability was also significantly elevated among IS patients with ES. Taken together, these findings suggest that seizures that occur in the early stage of IS are associated with poorer outcomes. In 7 of the studies examined, time-based classification of the PISS was not described (e.g., ES vs. LS). In addition, an exact time for the onset of the PISS was not reported. For these cases, a higher risk of mortality was also observed. Furthermore, cases involving LS were associated with a greater risk of disability. However, to date, an association between LS and mortality has not been reported. Therefore, additional prospective studies are needed to assess whether an association between LS and mortality exists.

In the present meta-analysis, the risk of death in patients with PISS was highest at discharge. While this risk subsequently decreased, it remained high up to 1 year after IS onset. Similarly, the risk of disability in patients with PISS was significantly higher at discharge, and then decreased 3 months after IS onset, despite remaining significant. According to the above-mentioned findings, PISS could influence both the short-term prognosis and long-term prognosis of patients with IS. In addition, the association between PISS and short-term prognosis is relatively more significant, partly because IS progresses rapidly in its early stages and it has a relatively high susceptibility to hyperexcitability.^[3,4]^ As a result, more serious pathologic changes can occur during the early stage of IS.^[4,28]^ In addition, the risk of death in patients with PISS was found to increase with age and was highest for patients older than 70 years. Thus, older patients with PISS potentially have a higher susceptibility to death. Taken together, these findings suggest that older individuals with PISS will have a poorer prognosis.

The latest guidelines for the early management of patients with IS did not provide an interpretation of the association between PISS and IS outcome.^[32]^ However, the present meta-analysis indicates that PISS are associated with increased risks of mortality and disability, and thus, clinicians should closely observe PISS events in patients with IS and prophylactic anticonvulsant drugs (PADs) may improve IS outcome. In studies of adult mice with PISS, the use of PADs such as diazepam, lorazepam, and phenytoin exhibited potential for improving mortality.^[3,4]^ However, we found that no relevant randomized control trial has assessed the efficacy and side effects of PADs in patients with IS. There are few data that have recently become available regarding the efficacy and safety of PADs in the treatment of PISS.^[33]^ Thus, the use of PADs after IS was not recommended by the latest guidelines,^[32]^ and there remains an urgent need for well-conducted randomized controlled trials to assess the efficacy and side effects of PADs after IS.

It is important to note that the present meta-analysis had several limitations. First, there was significant heterogeneity among the included studies, and meta-regression analysis was used to identify the sources of heterogeneity. Second, the quality of the included studies varied, and it was found that more high quality studies are needed. Third, the ways in which IS and PISS were treated among the included studies had the potential to affect the results of our meta-analysis. However, most of the included studies did not report treatment information, which could have contributed to information bias. Fourth, of the included studies, approximately 50% did not report whether they adjusted the potential confounders in their analysis of outcomes, and this may reduce the strength of our results despite the subgroup analyses that were conducted.

## 5 Conclusion

The results of the present study highlight that PISS, especially in the early stage of IS, are independently associated with higher risks of mortality and disability. Thus, increased awareness of early onset PISS, and their prevention when possible, by clinicians and researchers may improve the outcome of IS patients.

## Supplementary Material

Supplemental Digital Content
