# Supplementary material for: Association between seizures after ischemic stroke and stroke outcome: A systematic review and meta-analysis
Source: Medicine (Baltimore). 2016 Jul 8;95(27):e4117. doi: 10.1097/MD.0000000000004117 (PMC5058846; doi:10.1097/MD.0000000000004117)
Supplement: Supplemental Digital Content [file medi-95-e4117-s001.pdf]

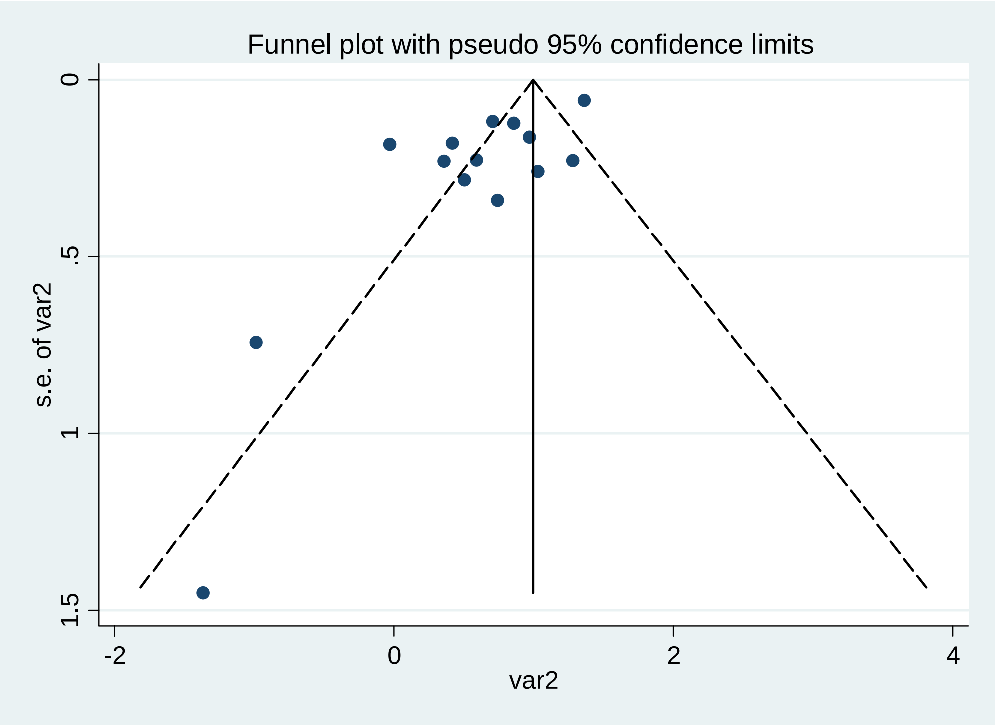

Figure S1. Funnel plot of the pooled relative ratio for those with PISS compared with those without PISS.

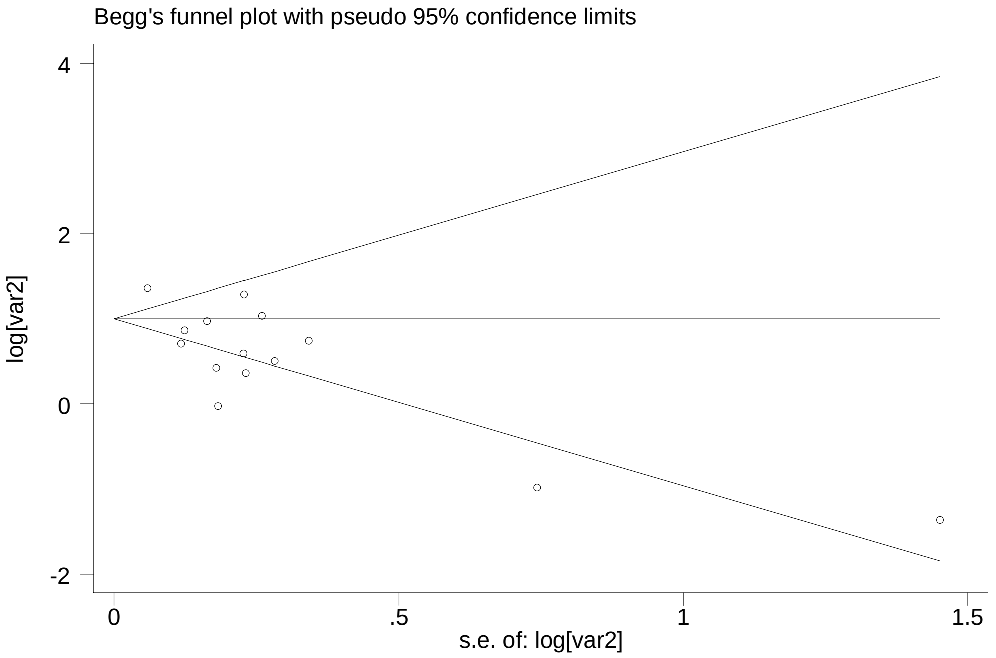

Figure S2. Begg's test for the pooled relative ratio for those with PISS compared with those without PISS: z = 0.22 (continuity corrected); Pr > |z| = 0.827 (continuity corrected).

Table S1 Characteristics of the studies included in the meta-analysis.

Author Publication year#	Region/country continent	Stroke diagnosis	Seizure or SE diagnosis	Sample size**	Age (Mean or median, years)	Outcomes	Comparison of BSS† between PISS and non-PISS groups	Comparison of comorbidities‡ between PISS and non-PISS groups	Adjusted for main confounders§
Bryndziar 2015[14]	USA/North America	Medical records	Self-report	Seizure:35; No seizure:454	77	Mortality	NA	No difference||	Yes
Kongbunkiat 2015[13]	Thailand/Asia	ICD-10	Self-report	Seizure:151; No seizure:81,910	Adults	Mortality	NA	NA	Yes
Huang 2014[25]	Canada/North America	Medical records	I LAE criteria	Seizure: 208; No seizure:10,053	72	Mortality; Disability*	More severe stroke in PISS group	No difference||	Yes
Hsu 2014[15]	China/Asia	Self-report	I LAE criteria	Seizure: 20; No seizure:158	8	Mortality	NA	No difference||	No
Hamidou 2013[16]	France/Europe	WHO criteria	I LAE criteria	Seizure:76; No seizure:2255	74	Mortality; Disability*	No difference||	No difference||	Yes
Arntz 2013[24]	Netherlands	By imaging evaluations	I LAE criteria	Seizure:54; No seizure: 482	40	Disability	More severe stroke in PISS group	Fewer hypertension and diabetes mellitus in PISS group	Yes
Couillard 2012[17]	Canada/North America	Self-report	I LAE criteria	Seizure:16; No seizure:384	74	Mortality	No difference||	More cardiac diseases in PISS group	No
Jung 2012[26]	Switzerland/Europe	By imaging evaluations	Self-report	Seizure:44; No seizure:761	Seizure:57; No seizure:63	Mortality; Disability*	More severe stroke in PISS group	No difference||	No
Burneo 2010[27]	Canada/North America	Medical records	I LAE criteria	Seizure:138; No seizure:4,889	71	Mortality; Disability*	More severe stroke in PISS group	Fewer diabetes mellitus in PISS group	No
Lee 2009[18]	China/Asia	ICD-9	I LAE criteria	Seizure:28; No seizure:47	Children	Mortality	NA	NA	No
Bateman 2007[19]	USA/North	ICD-9	ICD-9	Seizure:1681	Adults	Mortality	NA	Fewer hypertension and	Yes

WHO = World Health Organization; ILAE = International League Against Epilepsy; ICD = International Classification of Diseases; NA = not available; PISS = post-ischemic stroke seizures.

[*]Disability was assessed by modified Rankin Scale (mRS): disability was defined as mRS: 3 to 5.

[†]BSS is baseline stroke severity defined as stroke severity at hospital admission, and studies used National Institutes of Health Stroke Scale, Canadian Neurological Scale, or clinical findings to assess BSS at the hospital admission.

[‡]Comorbidity mainly included hypertension, diabetes mellitus, and cardiac diseases.

[§]Studies adjusted for main confounders [age, gender, life style (e.g., smoking, drinking), comorbidities, and BSS] in the analyses of study outcomes.

[||]No difference between PISS group and non-PISS group (≥ 0.05).

[#]Reference citations for the studies in this table are consistent with the reference citations in the main text.

[**]Seizure and no seizure refer to the groups that included or did not include patients experiencing PISS or PISS events, respectively.

Table S2. Quality assessment of the included studies*

Reference†	Is the exposed cohort representative?	Selection of the non-exposed cohort	Ascertainment of exposure	Demonstration that outcome of interest was not present at start of study	Comparability of important factors‡	Assessment of outcome	Follow-up period	Adequacy of follow up of cohorts	Total quality scores
Bryndziar 2015[14]	☆	☆	☆	—	☆	—	—	—	4
Kongbunkiat 2015[13]	☆	☆	☆	—	—	—	—	—	3
Huang 2014[25]	☆	☆	☆	☆	☆	☆	☆	☆	8
Hsu 2014[15]	☆	☆	☆	—	☆	—	☆	☆	6
Hamidou 2013[16]	☆	☆	☆	—	☆☆	☆	☆	☆	8
Arntz 2013[24]	☆	☆	☆	—	☆☆	☆	☆	☆	8
Couillard 2012[17]	—	☆	☆	☆	—	☆	—	☆	5
Jung 2012[26]	—	☆	—	☆	—	☆	☆	☆	5
Burneo 2010[27]	☆	☆	☆	☆	—	☆	☆	☆	7
Lee 2009[18]	☆	☆	☆	☆	☆	—	☆	☆	7
Bateman 2007[19]	☆	☆	☆	☆	—	—	—	—	4
Vernino 2003[20]	☆	☆	☆	—	—	☆	☆	☆	6
Arboix 2003[21]	☆	—	☆	☆	—	☆	—	☆	5
Bladin 2000[22]	☆	☆	☆	☆	☆☆	☆	☆	☆	9
Waterhouse 1998[23]	☆	☆	☆	—	☆	—	—	—	4

*The Newcastle-Ottawa Scale was used to assess study quality in this meta-analysis. High-quality studies received 8 stars.

† Reference citations for the studies in this table are consistent with the reference citations in the main text.

‡ A maximum of two stars could be awarded for this item. One star indicates an adjustment for age and two stars indicates an adjustment for gender.
